# Progress in immunotherapy for *Helicobacter pylori* infections

**DOI:** 10.3389/fcimb.2026.1839480

**Published:** 2026-06-12

**Authors:** Wane Zhong, Yan Yang, Ping Cao, Lian-xin Luo, Ke Wang

**Affiliations:** 1 Department of Gastroenterology, Ningbo NO.6 Hospital, Ningbo, China; 2 Department of Internal Medicine, Xianxiang Branch, The Second Hospital of Yinzhou District, Ningbo, China

**Keywords:** antibiotic therapy, *Helicobacter pylori*, *Helicobacter pylori* immune evasion, immunotherapy, pathogenic mechanism of *Helicobacter pylori*, synergistic treatment strategies

## Abstract

*Helicobacter pylori* is the most common cause of chronic gastric mucosal infections worldwide. The current global *H. pylori* infection rate is estimated at 50%. Untreated *H. pylori* colonization may lead to gastritis, peptic ulcers, and malignancies such as mucosa-associated lymphoid tissue (MALT) lymphoma and gastric cancer. *H. pylori* grows in the gastric mucosa by modulating host immunity, and can trigger a strong host immune response. The interaction between *H. pylori* and host immune defenses is complex, and the bacterium induces a gastric mucosal immunosuppressive microenvironment through multiple pathways to achieve colonization. This review analyzes the mechanisms involved in *H. pylori* pathogenic and immune evasion mechanisms, emphasizing the importance of identifying new therapeutic targets and developing effective treatment strategies to address the significant increase in the global prevalence of *H. pylori* antibiotic resistance. The review further discusses the synergistic status of *H. pylori* immunotherapy and antibiotic therapy and potential strategies for eradicating *H. pylori* infections.

## 1 Introduction

*Helicobacter pylori* is a bacterial pathogen that causes gastrointestinal diseases such as indigestion, gastritis, peptic ulcer, and stomach cancer. *H. pylori* eradication primarily relies on antibiotic treatment, primarily with amoxicillin. However, antibiotic resistance to *H. pylori* is rising in many parts of the world, especially to clarithromycin and levofloxacin. The success rate of *H. pylori* eradication is not satisfactory, often requiring the use of a combination of several antibiotics, which tends to disrupt the balance of intestinal flora and lead to further health complications. To address *H. pylori* antibiotic resistance, measures such as adjusting treatment strategies, optimizing antibiotic use, and introducing new drugs are required. Synergistic strategies of immunotherapy and antibiotic therapy offer more promise of therapeutic success with minimal disruption to healthy microbiomes (Fayed et al., 2023).

## 2 *H. pylori* pathogenesis and immune escape

### 2.1 Bacterial virulence factors and host immune response characteristics

**Figure 1:**
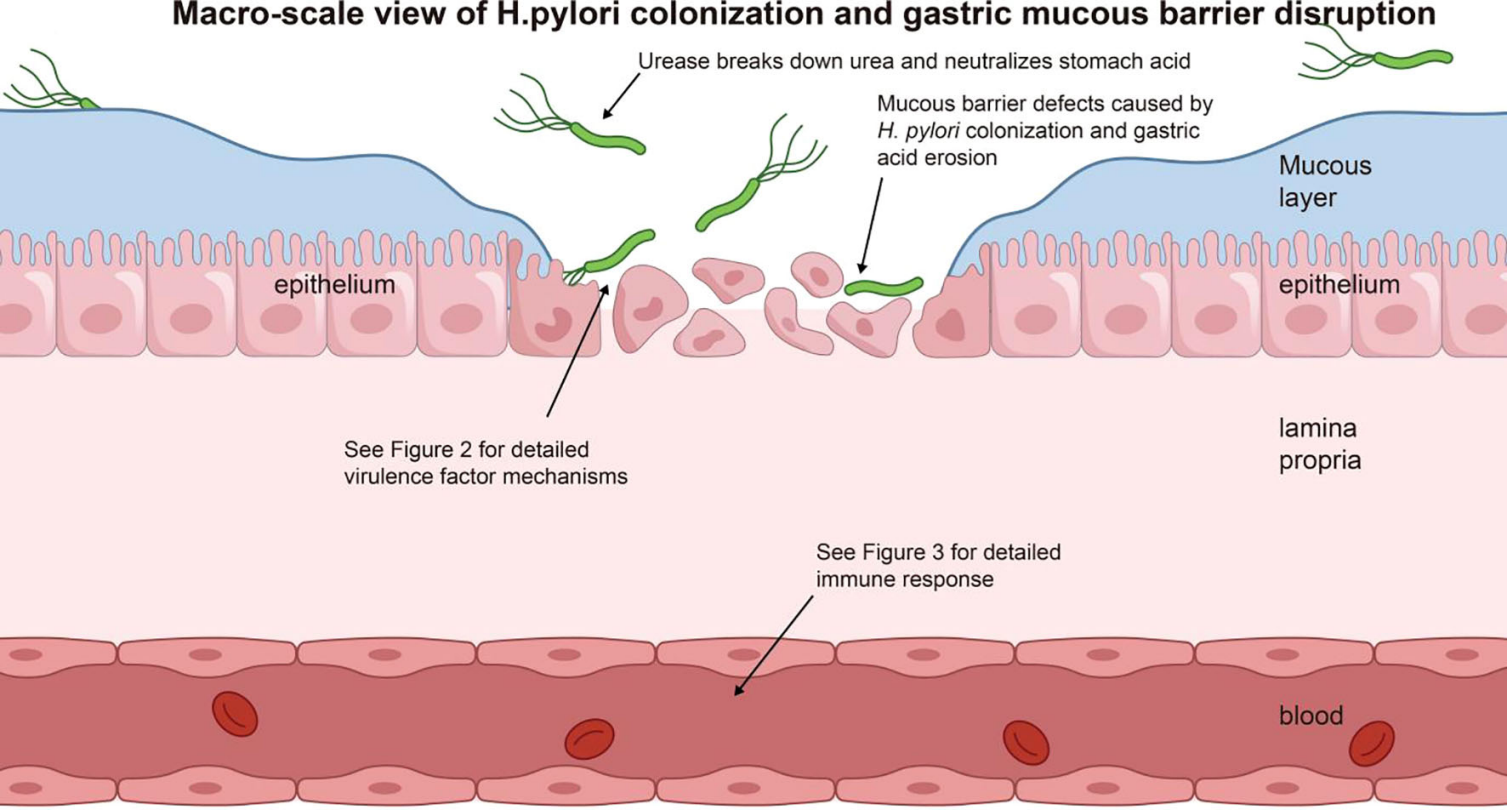
Macro-scale view of H.pylori colonization and gastric mucous barrier disruption.

**Figure 2:**
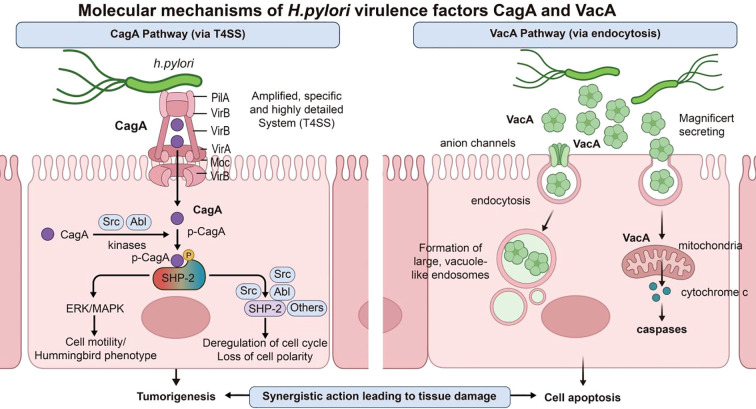
Molecular mechanisms of *H.pylori* virulence factors CagA and VacA.

**Figure 3:**
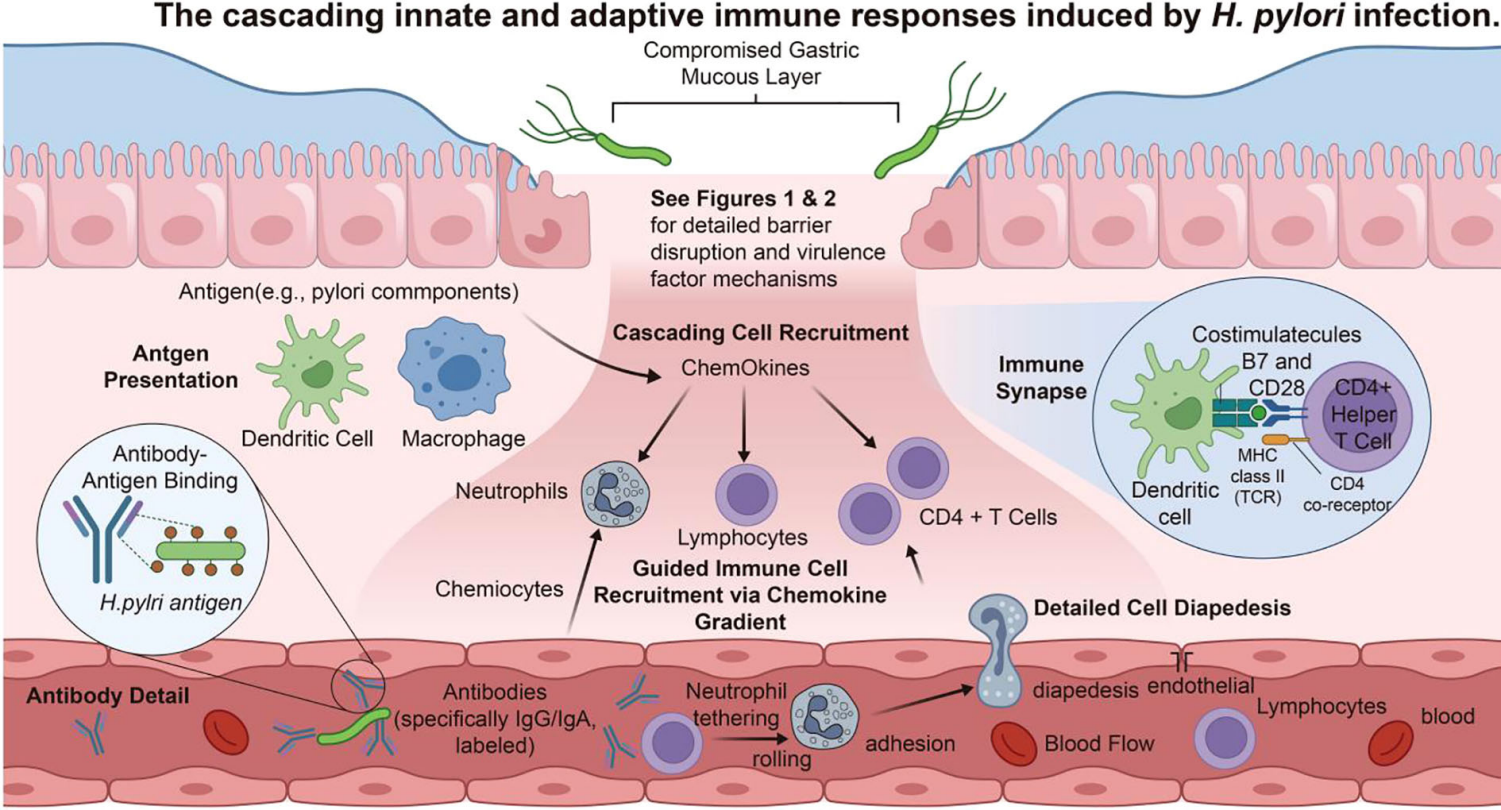
The cascading innate and adaptive immune responses induced by *H. pylori* infection.

As a class I carcinogen, *H. pylori*’s pathogenicity depends on the complex interaction between multiple virulence factors and the host immune system. Key virulence factors include cytotoxin-related gene A (*CagA*), which is injected into gastric epithelial cells through the type IV secretion system (T4SS), interfering with host cell signaling pathways, thereby promoting inflammatory responses and carcinogenesis (Cui et al., 2023; Duan et al., 2025),; and vacuole toxin A (VacA), which induces host cell vacuolation and inhibits T cell function (Cui et al., 2023), **Figures 1**, **2**, **3**. As the first line of defense against infection, Toll-like receptors (TLRs) of gastric epithelial cells recognize bacterial components; however, *H. pylori* evades recognition by TLR4 and TLR5 by modifying the lipopolysaccharide (LPS) structure and specific flagellin sequences, weakening the innate immune response (Pachathundikandi et al., 2023; Zhang et al., 2024). In addition, *H. pylori* metabolites can exacerbate gastritis by interacting with C-type lectin receptors after host cholesterol modification, further promoting T cell activation by activating innate immune cells (Nagata et al., 2021).

### 2.2 Mechanisms involved in induction of the immunosuppressive microenvironment

*H. pylori* infection can induce the formation of an immunosuppressive microenvironment in the gastric mucosa. This environment is characterized by immune escape phenomena such as T cell depletion and deficient antigen presentation, which ultimately leads to chronic persistent infection (Pernitzsch et al., 2021). The core mechanisms involved include:

#### 2.2.1 Upregulation of programmed death ligand 1

Bacterial infections (especially CagA-positive strains) promote the expression of PD-L1 by activating molecules such as squalene epoxidase (SQLE), which inhibits T cell activity and induces immune tolerance after binding to T cell PD-1 (Liu et al., 2025b; Li et al., 2025b).

#### 2.2.2 yeloid cell function regulation

After *H. pylori* invades the gastric mucosa, it interacts with immune cells in the lamina propria. Macrophages play a key role in the inflammatory response, and *H. pylori* stimulates them to secrete a variety of inflammatory factors such as interleukins (IL)-1β, IL-6, tumor necrosis factor (TNF)-α, and other pro-inflammatory factors. These factors induce regulatory T cell (Treg) expansion, inhibit effector T cell function, and lead to chronic damage to the gastric mucosa (Wei et al., 2024).

#### 2.2.3 Immune escape molecular networks

In addition to PD-L1, bacterial outer membrane vesicles (OMVs) act as virulence factor vectors, transmit immunosuppressive signals, and may interfere with host immune clearance by regulating autophagy pathways (Nabavi-Rad et al., 2023).

### 2.3 Antibiotic resistance and associated molecular pathways

The rate of drug resistance to *H. pylori* has shown a marked upward trend, with clarithromycin resistance reaching 92.1% in the Asia-Pacific region and levofloxacin resistance rates reaching 65.7% in Africa. Amoxicillin has a drug resistance rate of less than 2% in most countries, but more than 90% in some African countries. The rate of resistance to metronidazole is generally high, with the highest in Africa, reaching 100% (Schulz et al., 2025) (**Table 1**). *H. pylori* resistance is mainly mediated by gene mutations and adaptive mechanisms.

**Table 1:** *H. pylori* antibiotic resistance rates (%) in regions and countries worldwide (%) (data from 2018 to 2023).

Region and Country	Clarithromycin	Levofloxacin	Metronidazole	Amoxicillin
Region				
Africa	13.6–66.7	20–65.7	62.7–100	97.1
North America	16.7–19	42.6	29.3–35	1.1
South America	14.4–31.3	13.5–29	54	2.7
Asia Pacific	7.7–92.1	3.3–65.6	4.2–81.7	0–50
Europe	12–22.4	13–20.3	17–62.4	0–3.5
Country				
Vietnam	61.8–92.1	31.6–41.8	14.5–76.3	7.2–50
Democratic Republic of the Congo	23.5	65.7	90.2	34.3
Nigeria	25	ND	100	30–90.8
Egypt	40–52.8	20	100	81.9–95

#### 2.3.1 Target gene mutations

Clarithromycin resistance is associated with V-point mutations in the 23S rRNA domain (e.g., A2143G); Levofloxacin resistance is caused by *gyrA* gene mutations (such as N87K, D91G/Y); Metronidazole resistance involves inactivating mutations in oxygen-insensitive NADPH nitroreductase (RdxA) and nicotinamide nucleotide hydrogenase (FrxA) (Zhang et al., 2025a).

#### 2.3.2 Synergy between biofilm formation and immune escape

Bacteria form biofilm in the gastric mucosa, reduce antibiotic permeability, and decrease immune cell infiltration by enhancing immunosuppressive mechanisms such as PD-L1 expression (Liu et al., 2025b; Zhang et al., 2025a; Li et al., 2025b), further weakening the effectiveness of antibiotics.

#### 2.3.3 Strain heterogeneity and geographical variations:

During long-term colonization of its human host, *H. pylori* disturbs the gastric mucosa, leading to ulcers and gastric cancer. Multiple *H. pylori* virulence factors have been identified, showing extensive geographical differences. Regional strains such as the “Hardy” ecotype exhibit different drug-resistance profiles and immune escape owing to genomic differences, which affects the design of combination treatment strategies (Tourrette et al., 2024).

## 3 MImmunotherapeutic strategies: research progress

The development of effective immunotherapeutic strategies for *H. pylori* infection is critical for overcoming antibiotic resistance and improving eradication rates. Current progress is mainly focused on four major areas: vaccine development, immune checkpoint regulation, cytokine-directed intervention, and immunomodulation (**Table 2**).

**Table 2:** Immunotherapeutic strategies for *H. pylori* infection.

Field direction	Specific methods	Goal	References
Vaccine development	1. Targeting multiple virulence factors (such as LPS, CagA, VacA, adhesin, etc.). 2. Optimize the delivery system.	Prevent and treat *H. pylori* infection to reduce the risk of stomach cancer.	Liu et al. (2025a) and He et al. (2025)
Immune checkpoint regulation	Programmed death ligand 1 (PD-L1) inhibitors are used to block bacterial colonization.	Reduces the burden of *H. pylori* infection and inhibits pathogenicity.	Go et al. (2021)
Cytokine-directed intervention	1. Enhance Th1/Th17 response and T cell function. 2. Targeted metabolite intervention	Restore immune monitoring, eliminate infection, and reduce inflammatory responses.	Liu et al. (2025a)
Immunomodulators	1. Modulate T/B lymphocyte proliferation or surface receptor expression (e.g., increasing the CD4+/CD8+ ratio). 2.Inhibit inflammatory factors; promote the secretion of anti-inflammatory factors.	Enhance immune function, thereby enhancing the efficacy of antibiotics in eradicating *H. pylori*.	Guo et al. (2021) and Zhong et al. (2025)
Gastrointestinal Microecological Regulation	Supplementation with probiotics can strengthen the intestinal barrier; probiotic bacteria can adhere to the mucosal surface to prevent pathogen colonization, compete for nutrients and receptor sites, produce antimicrobial substances, and regulate the host’s immune response.	Restoring the disrupted gut microbiota and enhancing host immunity	Zhou et al. (2025)

### 3.1 Vaccine development: antigen target selection and delivery systems

The core challenges in vaccine development include insufficient immunogenicity of existing antigens and the lack of safe and effective adjuvants. *H. pylori* achieves immune escape by modifying LPS structures, which provides an important target for antigen design (Liu et al., 2025a). To enhance the immune response, novel multiantigen vaccines are rationally designed to improve protective efficacy by targeting multiple virulence factors (Chen et al., 2025). Although no vaccine has been available for more than 30 years, synthetic lipopeptide vaccines, such as Pam Cys-modified Hp4 and Hp10, successfully induce mucosal and T-cell immune responses by mimicking native antigenic epitopes and show considerable preventive effects in animal models (Xue et al., 2023). Bifunctional adjuvanted vaccines, such as compounds based on the 1,3,4-oxadiazole structure, further enhance immunogenicity by optimizing the adjuvant–antigen combination (Salem et al., 2025). In terms of delivery systems, oral microneedle vaccines can overcome mucosal barrier and immune tolerance, stimulate strong systemic and mucosal immunity without relying on additional adjuvant, and significantly reduce the infection burden in mice (He et al., 2025). Outer membrane vesicle (OMV) vector vaccines can induce a mixed Th1/Th2/Th17 immune response (dominated by Th17) and provide effective protection (Liu et al., 2025a). These advances mark notable breakthroughs in multicomponent vaccine design and novel delivery technologies (Chen et al., 2025).

### 3.2 Application of immune checkpoint modulation in infection control

*H. pylori* mediates immune escape through upregulation of programmed death ligand 1 (PD-L1) of gastric mucosal epithelial cells, promoting apoptosis and regulatory T cell (Treg) differentiation, leading to chronic infection (Li et al., 2025b). Preclinical studies have shown that blocking this pathway with PD-L1 antibodies or constructing a PD-L1 deficiency model through bone marrow transplantation can significantly reduce gastritis symptoms and reduce bacterial colonization levels in infected mice (Go et al., 2021)]. Dendritic cells (DCs) mediate PD-1/PD-L1 pathway activation: after infection, PD-L1-expressing DCs colocalize with T cells in the gastric mucosa, and their number is positively correlated with the degree of infection. Mouse models with DC clearance, such as Flt3 or Zbtb46-DTR mice, exhibited more severe gastritis and T-cell/neutrophil infiltration (Go et al., 2021). Based on this, novel PD-L1 nanoantagonists (such as BT@CPA) have been developed, which can achieve photothermal-chemo-immune synergy by specifically trapping bacteria, triggering chemotherapy, and modulating antimicrobial immunity, thus providing a new strategy for overcoming the immunosuppressive microenvironment (Li et al., 2025b). In addition, the virulence factor CagA can promote PD-L1 expression by upregulating squalene epoxase (SQLE), further revealing the association between immune checkpoints and bacterial oncogenic mechanisms (Liu et al., 2025a).

### 3.3 Cytokine-directed immune intervention strategies

*H. pylori* infection leads to immune dysfunction in the gastric ecosystem, which manifests as impaired antigen presentation and phagocytosis, extensive low reactivity of T cells, and limited clonal expansion, and the presence of T cell populations with high expression of HLA-DR and CTLA4 in infected gastric tissues; these cells may be involved in immune escape (Hu et al., 2025). Regarding intervention strategies, recombinant vaccines based on OMVs activate a combined Th1/Th2/Th17 immune response (predominantly Th17) and significantly reduce infection burden by enhancing cytokine secretion (e.g., IL-17) and antibody production (Liu et al., 2025a). In addition, *H. pylori* metabolites (e.g., cholesterol modifiers) can exacerbate gastritis by activating immune pathways, suggesting targeted metabolism-immune interactions as a potential intervention direction (Pachathundikandi et al., 2023). However, further research on targeted regulation of specific cytokines, especially how to reverse T cell depletion and restore immune surveillance, is critical (Hu et al., 2025).

### 3.4 Immunomodulation

Immunomodulators, such as immunoglobulins, spleen aminopeptides, probiotics, and vitamin D, can modulate the body’s immune function, enhance the body’s disease resistance, and exert an inhibitory effect on *H. pylori*. They can be used for patients with recurrent *H. pylori* infection. Studies have shown that dietary administration of anti- *H. pylori* immunoglobulin Y (anti-Hp IgY) significantly improved eradication rates and alleviated clinical symptoms in patients (Guo et al., 2021). A single-center, randomized, controlled study found that including treatment with egg yolk antibody to the bismuth quadruple therapy increased eradication rates and reduced adverse reactions (Cheng et al., 2023). The administration of spleen aminopeptide combined with standard quadruple therapy in the treatment of *H. pylori* infection significantly increases CD4+, CD3+, and CD4+/CD8+ levels, while those of CD8+ are significantly reduced, thereby improving the therapeutic effect (Zhong et al., 2025).

### 3.5 Potential therapeutic strategies for restoring the microbiome

Traditional antibiotic eradication therapy often induces marked alterations in the composition of the gut microbiota, leading to dysbiosis, impaired intestinal barrier function, and increased susceptibility to secondary infections (Saleem and Howden, 2020). To address these challenges, microbiota-protective strategies have been proposed. Adjunctive therapies such as probiotics, prebiotics, or fecal microbiota transplantation (FMT) can accelerate the restoration of microbial balance and improve clinical outcomes (Khoruts, 2018; Hrncir, 2022). Probiotics have been widely used as adjunctive therapy for *H. pylori* eradication. A meta-analysis of 45 randomized controlled trials involving approximately 7,000 patients demonstrated that probiotic supplements increased the intestinal eradication rate by approximately 13% and significantly reduced common gastrointestinal adverse effects such as diarrhea, nausea, and bloating (Lü et al., 2016). Probiotics reduce the risk of *H. pylori* infection by enhancing immune function, strengthening the intestinal barrier, and balancing the gut microbiota (Zhou et al., 2025).

## 4 Synergistic mechanism of combination therapy

### 4.1 Molecular basis by which immunomodulation enhances antibiotic sensitivity

*H. pylori* induces apoptosis and regulatory T cell (Treg) differentiation by upregulating PD-L1 expression in gastric mucosal epithelial cells, forming an immunosuppressive microenvironment to evade immune clearance, which leads to chronic infection (Go et al., 2021)]. Immunotherapeutic strategies can reverse bacterial-mediated immunosuppressive states by targeting immune checkpoints such as PD-L1. For example, PD-L1 nanoantagonist (BT@CPA) can specifically bind to *H. pylori*, triggering the release of chemotherapeutic drugs through mild photothermal therapy while activating the host antimicrobial immune response; this significantly enhances the killing effect of antibiotics against bacteria (Kamankesh et al., 2024). In addition, fucoidan (FU)-modified nanoparticles (FU/ML-LA/EB NPs) exhibit synergistic clearance against multidrug-resistant strains (MDRs) by promoting autophagy clearance and synergistic antibiotic penetration of biofilms (Zou et al., 2022; Yin et al., 2025). These mechanisms suggest that immunomodulation can restore host immune surveillance and weaken the bacterial resistance barrier, thereby enhancing antibiotic sensitivity.

### 4.2 Inhibitory effects of antibiotics on bacterial immune escape ability

Antibiotics directly kill bacteria, and disrupt immune escape-related molecular pathways of *H. pylori*. Studies have shown that *H. pylori* evades host immune recognition by modifying LPS structure, while antibiotics (e.g., clarithromycin) can interfere with the expression of LPS synthesis-related genes and weaken *H. pylori* immune escape ability (Pernitzsch et al., 2021; Zhang et al., 2025a). In addition, antibiotics can inhibit the secretion of bacterial virulence factors (such as VacA toxin) and reduce their inhibition of T cell proliferation, thereby restoring the local immune response (Go et al., 2021). In combination therapy, antibiotics reduce bacterial load, indirectly reduce the expression of immunosuppressive molecules such as PD-L1, block immune checkpoint inhibition between gastric epithelial cells and T cells, and enhance the clearance efficiency of immune cells against residual bacteria (Kamankesh et al., 2024).

### 4.3 Effect of combination therapy on biofilm penetration

The biofilm formed by *H. pylori*, being a key factor in its resistance to antibiotics, can hinder antibiotic penetration and protect bacteria from immune attack. Combination therapy reverses bacterial immune escape through immunomodulation and attenuates antibiotic resistance with virulence factor expression; furthermore, nanotechnology-based strategies enhance biofilm penetration, forming a multilevel synergistic mechanism to provide a new strategy for overcoming *H. pylori* resistance (Zhang et al., 2025; Saha et al., 2026). Combination therapy enhances penetration through the following mechanisms:

#### 4.3.1 Nanocarrier delivery system

Fucoidan-coated nanoparticles (FU/ML-LA/EB NPs) target the biofilm matrix and carry antibiotics to penetrate the mucus layer and biofilm, significantly increasing drug concentration at the site of infection (He et al., 2025). Photodynamic therapy nanosystems (e.g., RLs@T780TG) have mucus penetration and photosensitizer delivery functions, generate reactive oxygen species (ROS) under near-infrared light excitation, and synergize antibiotic degradation of biofilm structure (Qiao et al., 2024).

#### 4.3.2 Immunomodulatory enhanced penetration

Immune checkpoint inhibitors, such as anti-PD-L1 antibodies, activate macrophages to release ROS, which disrupts the stability of biofilms by regulating nickel-response regulators (NikR) and OMP2 protein expression (Wang et al., 2025). At the same time, activated immune cells (such as neutrophils) can secrete antimicrobial peptides that further synergize with antibiotics to penetrate deep biofilms (Gupta et al., 2023).

## 5 Resistance management and relapse control

The problem of drug resistance in *H. pylori* infection is becoming increasingly serious, significantly hindering the effectiveness of eradication treatment and increasing the risk of recurrence. The strategy of combining immunotherapy with antibiotics provides a new direction for resistance management and relapse control.

### 5.1 Inhibitory effect of combined strategies against resistant strains

Antibiotic monotherapy can induce drug resistance, whereas combination immunomodulator-antibiotic strategies can inhibit resistant strains through multitarget action. Studies have shown that immunomodulators (such as PD-L1 inhibitors) can reverse bacterial-mediated host immunosuppressive states, block the PD-L1 signaling pathway, restore T cell activity, enhance antibiotic sensitivity to drug-resistant strains, and promote bacterial clearance (Fan et al., 2024). In addition, nanomaterials such as inorganic nanoparticles, lipid-based nanoparticles, and polymeric nanoparticles exert effects such as disrupting bacterial cell membranes, controlling drug release, and overcoming antibiotic resistance (Meng et al., 2025). Non-antibiotic strategies such as photodynamic therapy (PDT) show potent inhibitory effects on multidrug-resistant strains (MDRs) by producing ROS to disrupt bacterial structure, with no side effects on healthy tissues (Im et al., 2021).

### 5.2 Mechanisms of maintenance of immune memory after treatment

Long-term immune memory is key to preventing relapse. *H. pylori* upregulates the expression of PD-L1 in gastric mucosal epithelial cells and induces T cells apoptosis and regulatory T cell (Treg) differentiation, achieving immune escape and hindering memory formation (Fan et al., 2024). In combination therapy, immunomodulatory components (such as nanovaccines or cytokine-directed therapies) can promote the activation of antigen-presenting cells, drive the Th1 immune response, and enhance effector T cell function (Koch et al., 2023). For example, multiantigen vaccines are designed to induce long-lasting antibody and memory T cell responses by activating the mucosal immune system (Zhang et al., 2025b). Animal models have confirmed that PD-L1 nanoantagonists (BT@CPA) combined with mild photothermal therapy can reshape the antimicrobial immune microenvironment and reduce relapse (Gupta et al., 2023)].

### 5.3 Novel molecular detection techniques for recurrence monitoring

Traditional tests, such as urea breath tests, offer low effectiveness in distinguishing between relapse and reinfection and cannot monitor resistance in real time. Innovative solutions based on molecular technology significantly improve monitoring accuracy. The immune-antibiotic combination strategy provides a multidimensional solution for drug resistance management and relapse control by synergistically inhibiting drug-resistant bacteria, maintaining immune memory, and enabling precise monitoring. The new molecular detection technologies include:

#### 5.3.1 Rapid detection of drug-resistance genes

Genotype susceptibility testing (G-AST) of gastric juice or fecal samples can directly detect mutations in 23S rRNA (clarithromycin resistance) and gyrA (levofloxacin resistance), shortening the detection period compared with phenotypic susceptibility testing (P-AST) (Lai et al., 2025a; Vasapolli et al., 2025).

#### 5.3.2 Non-invasive resistance profiling

Metagenomic sequencing technology can simultaneously track resistance genes (ARGs) and strain variants to assess gut microbiome perturbations and changes in the resistance gene pool after treatment (Wang et al., 2023).

#### 5.3.3 AI-assisted prediction

AI models that combine clinical data with drug resistance gene databases can predict individualized recurrence risk and optimize subsequent treatment options (Lai et al., 2025a; Dore et al., 2025).

## 6 Current challenges and controversies

### 6.1 Safety and long-term effects of immunotherapy

The safety of immunotherapy remains a core clinical concern. *H. pylori* can evade immune surveillance by upregulating the immune checkpoint molecule PD-L1, and immunomodulatory strategies targeting such pathways may trigger the risk of excessive immune activation or autoimmune response (Ramesh et al., 2025). In addition, although novel therapies such as photodynamic therapy, acoustic nanoplatforms, and targeted nanodrugs have shown efficient bactericidal capabilities in preclinical studies, their long-term biocompatibility, potential damage to the gastric mucosal barrier, and interference with commensal flora have not been fully evaluated (Luo et al., 2023). Traditional antibiotic treatment has been identified as causing gastrointestinal and vaginal dysbiosis (Wang et al., 2024a), whereas the long-term effects of immunotherapy on the host microecology still warrant verification through large-scale clinical studies (Kang et al., 2025).

### 6.2 Influence of regional strain differences on efficacy

Globally, genetic heterogeneity in *H. pylori* strains significantly influences treatment response. The rate of primary antibiotic resistance continues to rise in the Asia-Pacific region, with clarithromycin resistance and levofloxacin resistance rates at high levels (Hong et al., 2024; Schulz et al., 2025). Clarithromycin resistance is as high as 36.7% (95% CI: 30.0%–43.9%) in East Asian children (Ramezani et al., 2025), compared with 31.5% in some parts of the United States (Ho et al., 2022). Further, geographical differences in the distribution of strain virulence factors (e.g., CagA, VacA), lead to different immune escape mechanisms (Li et al., 2025a). This variability requires that treatment regimens be individually tailored based on local resistance surveillance data.

### 6.3 Cost-benefit analysis and health economics considerations

Combination therapies face multiple challenges from a health economics perspective. Although individualized treatment guided by susceptibility testing can improve eradication rates, it requires additional endoscopic biopsy (additional cost of 150–300 per case) and 5–7 days of detection (Lopes-de-Campos et al., 2022). Genetic testing, such as clarithromycin resistance mutation identification, is costly, limiting its application in resource-limited areas (Vasapolli et al., 2025). In contrast, empiric bismuth quadruple therapy (BQT) is used as a first-line regimen in most guidelines (Chey et al., 2024). The new quadruple regimen containing vonolasan has an eradication rate of 90.6% in phase III clinical trials in China, which is significantly higher than that of traditional PPI regimens, and its long-term cost-effectiveness warrants evaluation in combination with recurrence rate and adverse reaction management (Hou et al., 2024). In addition, dual therapy (e.g., vonolasan combined with high-dose amoxicillin) can only be used in patients who are not allergic to penicillin, and its widespread use may induce drug resistance, potentially undermining its cost-effectiveness advantage (Duan et al., 2023). Health systems need to weigh the cost of testing, the cost of treatment, and the long-term burden of the spread of drug-resistant bacteria (Zhou et al., 2022).

## 7 Future research directions

### 7.1 Screening platforms for novel immune-antibiotic combinations

The current widely used *H. pylori* antibiotic treatment regimens in clinical practice are facing increasingly severe challenges: the resistance rate continues to rise, eradication efficiency is decreasing year by year, and the damage to the intestinal microecology is becoming increasingly prominent. In this context, the development of new treatment strategies to overcome the limitations of antibiotic therapy has become a major issue requiring rapid resolution. High-throughput screening platforms to accelerate the development of immune-antibiotic synergistic therapies would represent an important advancement in this area. Key directions include:

#### 7.1.1 Nanodelivery system optimization

Development of gastric acid-responsive nanocarriers (e.g., lipid nanoparticles containing amoxicillin; Lopes-de-Campos et al., 2022) to improve antibiotic targeted delivery, while loading immunomodulators (e.g., PD-L1 antagonists; Gupta et al., 2023) to disrupt bacterial immune escape (Wang et al., 2024b).

#### 7.1.2 Photo/acoustic therapy integration

Constructing multifunctional nanoprobes (e.g., PtCu-PDA@AIPH@Fucoidan (Zhou et al., 2025), Ver-PLGA@Lecithin; Liu et al., 2024), combining sound/photodynamic therapy with antibiotics, enhancing biofilm penetration and removal ability of drug-resistant bacteria (Wang et al., 2022).

#### 7.1.3 Engineered bacterial drug delivery platform

CRISPR-Cas9 technology is used to construct engineered *H. pylori* strains (such as Hp-Ce6) to achieve *in situ* drug release and immune activation, providing a new strategy for synergistic tumor therapy (Zeng et al., 2024).

### 7.2 Microbiome-oriented personalized treatment strategies

*H. pylori* infection can disrupt the balance of the gastrointestinal microbiome, leading to alterations in the structure, diversity, and function of the microbiome in the stomach and gut. Although *H. pylori* eradication treatment can ameliorate gastric infections, it may also have short-term effects on the microbiome. Maintaining the balance of the gastrointestinal microbiome is of great importance for the prevention and treatment of *H. pylori*-related diseases, and probiotic-assisted therapy may be used to further optimize treatment and reduce adverse effects on the microbiome. To address the challenge of microbiome imbalance, research should focus on:

#### 7.2.1 Precise regulation of microbiota

Metagenomic sequencing was used to analyze the dynamic changes of the enterovirome after *H. pylori* eradication (Ansari and Yamaoka, 2022), identify protective microbiota markers (e.g., *Lactobacillus* spp. (Lai et al., 2025b), and design probiotic-antibiotic sequential therapy to maintain microecological balance (Duan et al., 2023; Han et al., 2025).

#### 7.2.2 Host-microbiota interaction mechanisms

Elucidating the immune cross-regulatory network of *H. pylori* and the gastric microbiome (e.g., non-*H. pylori*) (Chitas et al., 2024), and developing microbiome-directed immunomodulators (e.g., fucoidan nanocomplexes) to repair the mucosal barrier (Cao et al., 2025; ; Zhang et al., 2026).

#### 7.2.3 Personalized medication model

Based on regional strain differences and host genetic polymorphisms, a microbiome-guided dosing regimen is established to reduce the recurrence rate (Ho et al., 2022; Namikawa et al., 2025).

### 7.3 AI-assisted joint scheme optimization

Studies have shown that AI clinical decision-making systems based on reinforcement learning have brought the era of *H. pylori* root cure back to the era of 94% (Higgins et al., 2025). Artificial intelligence technology can recommend an optimal *H. pylori* eradication plan by comprehensively considering the patient’s age, gender, antibiotic allergy history, region, and medication status.

Efficacy prediction models.

Machine learning (e.g., random forests, XGBoost algorithms) is used to integrate clinical multiomics data (including antibiotic-resistance genes (Vasapolli et al., 2025) and host immune markers; Luo et al., 2025) to predict different combination regimens (e.g., dual/quadruple therapy; Qian et al., 2023; Han et al., 2023) and the risk of recurrence (Luo et al., 2025; Lai et al., 2025b).

Real-time monitoring of drug resistance.

A rapid detection platform for *H. pylori* resistance genes (Lai et al., 2025a) was developed to dynamically adjust antibiotic combinations by identifying resistance mutations (e.g., clarithromycin 23S rRNA point mutations; Vasapolli et al., 2025) in order to avoid empirical drug failure (Jaiswal et al., 2025)].

Multiobjective optimization algorithm.

Combining cost-benefit analysis and health economics parameters (Ma et al., 2024), a multiobjective decision-making model is constructed to optimize the immune-antibiotic dose ratio and duration of treatment (e.g., 14-day high-dose dual therapy; Hsu et al., 2023) to balance efficacy, safety, and healthcare costs (Ang et al., 2022; Iqbal et al., 2025).

## 8 Conclusions

*H. pylori* has a high infection rate worldwide and is an important pathogenic factor causing gastrointestinal diseases such as gastritis, peptic ulcers, and gastric cancer. *H. pylori* achieves immune tolerance and immune evasion through complex interactions with the host’s immune system via multiple virulence factors. Owing to the rising antibiotic resistance of *H. pylori* in many regions globally, the success rate of its eradication has gradually declined. The synergistic strategy of immunotherapy combined with antibiotic therapy offers a more promising approach for *H. pylori* eradication. The application of nanotechnology, microbiome-guided personalized treatment, artificial intelligence-assisted treatment plans, and safe and efficient immunomodulators will markedly improve the eradication rate of this bacterial pathogen.
